# Oncological prognosis and morbidity of hepatectomy in elderly patients with hepatocellular carcinoma: *a propensity score matching and multicentre study*

**DOI:** 10.1186/s12893-023-02230-0

**Published:** 2023-10-24

**Authors:** Chuan-Ming Wang, Zi-Xiang Chen, Ping-Chuan Ma, Jiang-Ming Chen, Dong Jiang, Xin-Yuan Hu, Fu-Xiao Ma, Hui Hou, Jin-Liang Ma, Xiao-Ping Geng, Fu-Bao Liu

**Affiliations:** 1 Department of General Surgery, The First Affiliated Hospital of Anhui Medical University, Anhui, 230022 China; 2 Department of General Surgery, The Second Affiliated Hospital of Anhui Medical University, Anhui, 230601 China; 3 Department of General Surgery, The First Affiliated Hospital of University of Science and Technology, Anhui, 230031 China

**Keywords:** Hepatocellular carcinoma, Hepatectomy, Elderly patients, Propensity score matching, Prognosis, Cancer-specific survival

## Abstract

**Purpose:**

With increasing life expectancy, the number of elderly patients (≥ 65 years) with hepatocellular carcinoma (HCC) has steadily increased. Hepatectomy remains the first-line treatment for HCC patients. However, the prognosis of hepatectomy for elderly patients with HCC remains unclear.

**Methods:**

Clinical and follow-up data from 1331 HCC patients who underwent surgery between 2008 and 2020 were retrospectively retrieved from a multicentre database. Patients were divided into elderly (≥ 65 years) and non-elderly (< 65 years) groups, and PSM was used to balance differences in the baseline characteristics. The postoperative major morbidity and cancer-specific survival (CSS) of the two groups were compared and the independent factors that were associated with the two study endpoints were identified by multivariable regression analysis.

**Results:**

Of the 1331 HCC patients enrolled in this study, 363 (27.27%) were elderly, while 968 (72.73%) were not. After PSM, 334 matched samples were obtained. In the propensity score matching (PSM) cohort, a higher rate of major morbidity was found in elderly patients (P = 0.040) but the CSS was similar in the two groups (P = 0.087). Multivariate analysis revealed that elderly age was not an independent risk factor associated with high rates of major morbidity (P = 0.117) or poor CSS (P = 0.873). The 1-, 3- and 5-year CSS rates in the elderly and non-elderly groups were 91.0% versus 86.2%, 71.3% versus 68.8% and 55.9% versus 58.0%, respectively. Preoperative alpha fetoprotein (AFP) level, Child‒Pugh grade, intraoperative blood transfusion, extended hemi hepatectomy, and tumour diameter could affect the postoperative major morbidity and preoperative AFP level, cirrhosis, Child‒Pugh grade, macrovascular invasion, microvascular invasion (MVI), satellite nodules, and tumor diameter were independently and significantly associated with CSS.

**Conclusion:**

Age itself had no significant effect on the prognosis of elderly patients with HCC after hepatectomy. Hepatectomy can be safely performed in elderly patients after cautious perioperative management.

**Supplementary Information:**

The online version contains supplementary material available at 10.1186/s12893-023-02230-0.

## Introduction

Hepatocellular carcinoma (HCC) ranks third among causes of cancer-related deaths worldwide [1] and ranks second in men [2]. HCC has a poor prognosis, with a 1-year survival rate of less than 50% after disease onset [3]. The incidence of HCC has increased dramatically in the last three decades and the number of HCC cases is expected to continue increasing in the next 20 years [4]. With the rapid development of medical technology and increased access to health care, as well as improved human life expectancy, the ageing of the population is an inevitable phenomenon [5]. Ageing is inherently a common risk factor for the development of malignant tumours, and HCC has a higher specific incidence among elderly patients worldwide [6]. Studies have shown that the development of HCC is closely associated with ageing [7], and the number of elderly patients with HCC is expected to increase considerably [8].

Currently, partial hepatectomy remains the preferred treatment for selected patients with HCC [9]. Previous studies have revealed that hepatectomy can also be safely performed in elderly HCC patients [10, 11]. However, the potential benefits among this population are not well elucidated. In addition, identification of risk factors associated with tumour prognosis in elderly patients, more accurate assessment of surgical risk, and more comprehensive perioperative management are essential for reducing the probability of serious postoperative complications and improving long-term prognosis [12]. Studies have shown that age is strongly associated with the long-term prognosis of some tumours. For example, in gastric [13], colorectal [14] and lung [15] cancers, the long-term prognosis is worse in younger patients, while in prostate [16] and thyroid [17] cancers, the prognosis is worse in older patients. Age can act as an independent factor affecting long-term prognosis. There are few studies on the correlation between age and long-term prognosis in HCC patients treated with hepatectomy, and the findings are varied and contradictory [18]. Some studies have concluded that elderly patients have similar long-term outcomes as younger patients, and sometimes elderly patients could even perform better in recurrence [19, 20], however these studies have not revealed the relationship between old age and prognosis. Another study concluded that young (≤ 35 years of age) HCC patients had a higher recurrence rate than elderly patients, but overall survival was similar in both young and old patients. This may be due to the higher incidence of chronic disease and nononcologic death in elderly patients, but the study neglected the relatively large middle-aged patient population (> 35 years and < 65 years of age) [21]. Such studies also have the disadvantage of being mostly single-centre studies with small sample sizes, wherein some selection bias is inevitable.

This study aimed to use a multicentre database to compare cancer-specific survival (CSS), and the probability of serious postoperative complications in elderly versus nonelderly HCC patients treated with R0 hepatectomy, and to use the PSM method to balance baseline characteristics between the two datasets. We defined cancer-specific survival as the time from diagnosis of HCC to death due to HCC.

## Methods

### Study population

The data used in this study were collected between 2008 and 2020 from patients who underwent curative-intent hepatectomy for HCC at the First, and Second Affiliated Hospital of Anhui Medical University and, the First Affiliated Hospital of University of Science and Technology of China, which are three high-volume hepato-pancreato-biliary surgery centres. The study was conducted in accordance with the Declaration of Helsinki and Ethical Guidelines for Clinical Studies (No. P-2021-1230). This was a retrospective clinical study, and the family members of the participants were informed of the scientific use of their clinical data at the time of the preoperative interview and were not required to provide a separate written informed consent. Radical hepatectomy was defined as the absence of residual tumour on intraoperative visual or ultrasound examination and the absence of residual tumour on postoperative microscopic pathological examination of the resection margins. According to the World Health Organization, most developed countries have adopted the definition of ≥ 65 years as elderly individuals, that is, elderly patients were defined as ≥ 65 years of age at the time of the procedure and nonelderly patients were defined as < 65 years of age at the time of the procedure. The exclusion criteria for this study were R1 or R2 resection (palliative surgery), recurrent HCC, preoperative adjuvant therapy, combined hepatocellular-cholangiocarcinoma or other malignancies, and a significant lack of clinical or follow-up data.

### Data collection

All operations were performed by experienced liver surgeons. The required clinical data were divided into three parts, namely preoperative examination, surgical variables, and tumour characteristics. Preoperative examinations included age, sex, American Society of Anaesthesiologists (ASA) score, body mass index (BMI), comorbidity, alpha-fetoprotein (AFP), alanine aminotransferase (ALT), aspartate transaminase (AST), hepatitis B virus (HBV) infection, cirrhosis, portal hypertension, Barcelona Clinic Liver Cancer (BCLC) staging and Child‒Pugh grade. Surgical variables included operative approach, margin width, blood loss, intraoperative blood transfusion, operation duration, anatomical hepatectomy, extended hemi hepatectomy and occlusion. Tumour characteristics included macrovascular invasion, microvascular invasion (MVI), satellite nodules, tumor diameter, tumour differentiation and tumour envelope. Cirrhosis was confirmed by preoperative imaging, intraoperative exploration, or postoperative pathology. Anatomical hepatectomy was defined as hepatectomy with the systematic removal of a hepatic segment, and segmentation was based on liver function [22]. The degree of extended hemi-hepatectomy was defined as more than a hemihepatic lobe that was resected [23]. The ASA scores of all patients were determined by a dedicated anaesthesiologist before surgery based on the preoperative findings, and all the ratings were accurate and reliable.

### Follow-up

Follow-up was performed primarily through outpatient clinic visits or by telephone. The follow-up included monthly reexamination for 3 months after surgery, once every 3 months at 4–24 months after surgery, every 6 months at 2–5 years after surgery, and annual visits starting at 5 years after surgery. Follow-up evaluation included assessment for postoperative complications, AFP, liver function tests, and imaging tests. Ultrasound, computed tomography (CT), or MRI were considered acceptable but should have included an enhanced CT or MRI within one year. Three follow-up endpoints were defined: presence of serious complications, tumour recurrence, and patient death. Major morbidity was defined as postoperative complications with Clavien‒Dindo grade ≥ 3 related to the procedure, including perioperative complications or readmission due to complications. Tumour recurrence must be confirmed by enhanced CT, MRI, positron emission tomography (PET)-CT, or needle biopsy, with detailed documentation of the time of recurrence, AFP value, and site of recurrence. It is essential to confirm whether patient death is cancer specific. Noncancer specific death includes death due to liver failure grade-related complications, gastrointestinal bleeding, unexplained death due to age, and other tumour induced death.

### Statistical analysis

Patients in the elderly group and non-elderly group were matched by using the PSM method as described by Rosenbaum and Rubin [24, 25]. The propensity score for an individual was estimated using logistic regression [26] and the co-variables in the regression model included sex, ASA score, BMI, comorbidity, AFP, ALT, AST, HBV infection, cirrhosis, portal hypertension, BCLC staging, Child‒Pugh grade, operative approach, margin width, blood loss, intraoperative blood transfusion, operation duration, anatomical hepatectomy, extended hemi hepatectomy, occlusion, macrovascular invasion, MVI, satellite nodules, tumor diameter, tumour differentiation and tumour envelope. We applied 1:1 nearest neighbor matching without replacement in order to ensure that conditional bias was minimized. The nearest neighbor matching was based on a greedy matching algorithm [27], which matched each unit in the elderly group to a unit in the non-elderly group that had the closest propensity score. The appropriateness of matching was assessed by comparing the standardized differences in covariate means for continuous variables and differences in covariate means for dichotomous variables for the matched and unmatched samples. Small absolute values in standardized differences (< 10%) were assumed to support the assumption of balance between the treatment groups. A calliper width of 0.020 resulted in the best trade-off between homogeneity and retained sample size. More stringent calliper was also attempted but 0.020 gave the best matching model.

Categorical variables are expressed as numbers or proportions, and continuous variables are expressed as the mean ± standard deviation or median (range). The t test was used to compare continuous variables, and the chi-square test was used to compare categorical variables. Survival analysis was performed using the Kaplan‒Meier method, and the log-rank test was used to analyse differences between the two groups. Univariate and multivariate analysis was used to identify prognostic variables associated with CSS and major morbidity. All statistical analyses were conducted using SPSS 23.0 (SPSS Inc, Armonk, NY, USA) and R 4.2.0 (http://www.r-project.org/, R Development Core Team). A P value < 0.05 was considered to indicate a significant difference in a 2-tailed test.

## Results

### Clinicopathological characteristics

**Fig. 1:**
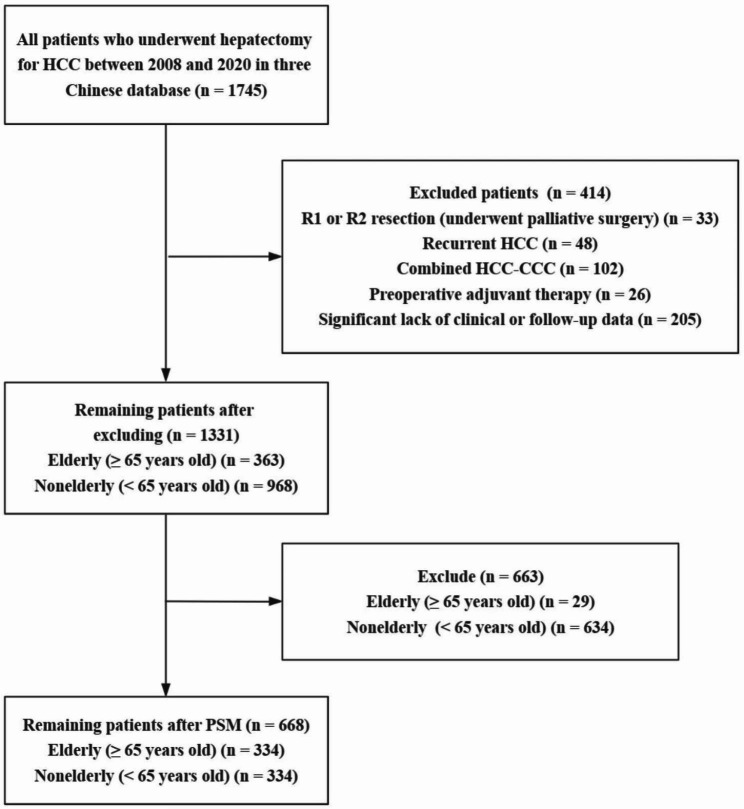
Selection of the study population. HCC, hepatocellular carcinoma; HCC-CCC, hepatocellular-cholangiocarcinoma

**Fig. 2:**
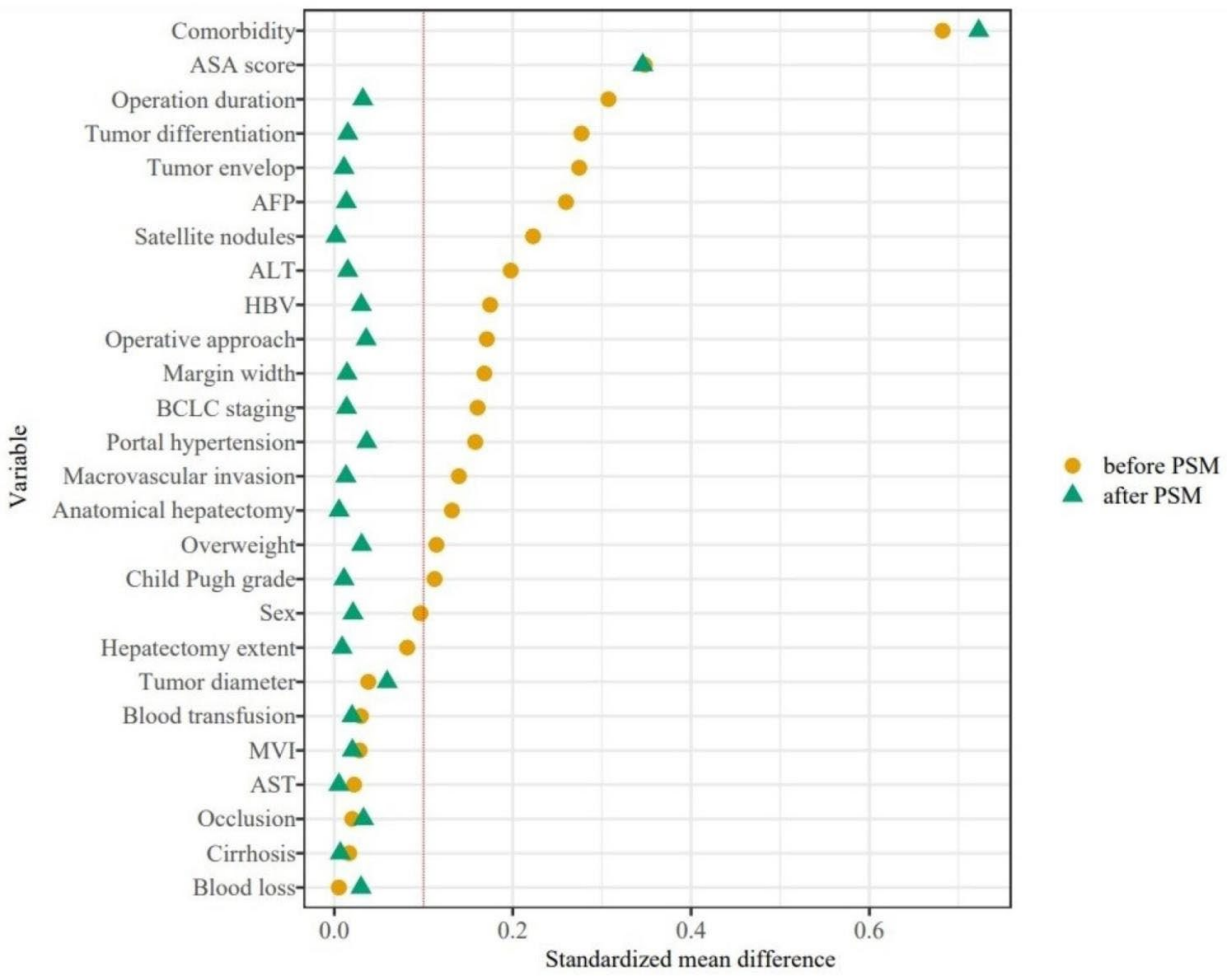
Absolute standardized difference before and after PSM

Using the inclusion and exclusion criteria, a total of 1331 patients were enrolled in the present study, including 363 (27.27%) elderly patients with a mean age of 70.38 ± 4.46 years (*P* < 0.001), and 968 (72.73%) nonelderly patients with a mean age of 51.55 ± 8.60 years (*P* < 0.001) (Fig. 1). Data on the 334 matched groups of elderly and nonelderly patients were obtained by PSM. In the matched groups, the mean age of elderly patients was 70.25 ± 4.47 years (*P* < 0.001), and that of nonelderly patients was 52.16 ± 8.66 years (*P* < 0.001). The clinical and pathological characteristics and surgical variables between the two groups before and after PSM are showed in Table 1. After PSM, there were no significant differences in any liver or tumour-related variables between the nonelderly and elderly patients (all *P* > 0.200), except for the ASA score (34.13% vs. 20.36%, *P* < 0.001) and comorbidities (48.50% vs. 14.67%, *P* < 0.001). This is consistent with our common sense of life, as the ASA score and comorbidities are intrinsic variables that are related to age. The love plots of the absolute standardized differences before and after PSM show that all covariates are below the threshold of 10% except the ASA score and comorbidities. This means that propensity score matching has balanced the treatment and control groups regarding these covariates (Fig. 2).

**Table 1:** The clinical, operative and pathological characteristics between the elderly and nonelderly group before and after propensity score matching

Characteristics	Before PSM(n = 1331)		*P*	After PSM(n = 668)		*P*
	Elderly	Nonelderly		Elderly	Nonelderly	
	n = 363	n = 968		**n = 334**	n = 334	
Age #	70.38 (4.46)	51.55 (8.56)	< 0.001	70.25 (4.47)	52.16 (8.66)	< 0.001
Male sex	298 (82.09)	829 (85.64)	0.130	274 (82.04)	277 (82.93)	0.839
ASA score > 2	124 (34.16)	184 (19.01)	< 0.001	114 (34.13)	68 (20.30)	< 0.001
Overweight	107 (29.48)	337 (34.81)	0.076	97 (29.04)	95 (28.44)	0.932
Comorbidity	177 (48.76)	177 (18.29)	< 0.001	162 (48.50)	49 (14.67)	< 0.001
AFP > 400 µg/l	92 (25.34)	361 (37.29)	< 0.001	91 (27.25)	88 (26.35)	0.861
ALT > 40 U/L	117 (32.23)	404 (41.74)	0.002	115 (34.43)	111 (33.23)	0.806
AST > 40 U/L	163 (44.90)	424 (43.80)	0.765	147 (44.01)	147 (44.01)	0.999
HBV infection	300 (82.64)	859 (88.74)	0.004	283 (84.73)	285 (85.33)	0.914
Cirrhosis	165 (45.45)	432 (44.63)	0.835	148 (44.31)	147 (44.01)	0.999
Portal hypertension	113 (31.13)	374 (38.64)	0.014	108 (32.34)	104 (31.14)	0.803
Child‒Pugh grade B	11 (3.03)	51 (5.27)	0.114	11 (3.29)	10 (2.99)	0.999
BCLC staging B/C	109 (30.03)	364 (37.60)	0.012	98 (29.34)	101 (30.24)	0.866
**Intraoperative variables**						
Open operative approach	283 (77.96)	819 (84.61)	0.005	264 (79.04)	268 (80.24)	0.773
Margin width < 1 cm	63 (17.36)	111 (11.47)	0.006	49 (14.67)	50 (14.97)	0.999
Blood loss > 600ml	29 (7.99)	76 (7.85)	0.999	25 (7.49)	29 (8.68)	0.670
Intraoperative blood transfusion	84 (23.14)	212 (21.90)	0.682	79 (23.65)	85 (25.45)	0.653
Operation duration > 180 min	234 (64.46)	757 (78.20)	< 0.001	226 (67.66)	224 (67.07)	0.934
Anatomical hepatectomy	190 (52.34)	443 (45.76)	0.038	170 (50.90)	166 (49.70)	0.816
Extended hemihepatectomy	56 (15.43)	179 (18.49)	0.220	53 (15.87)	61 (18.26)	0.472
Occlusion	200 (55.10)	543 (56.10)	0.791	183 (54.79)	196 (58.68)	0.349
**Tumor variables**						
Macrovascular invasion	25 (6.89)	105 (10.85)	0.039	24 (7.19)	18 (5.39)	0.425
MVI	110 (30.30)	306 (31.61)	0.695	104 (31.14)	87 (26.05)	0.171
Satellite nodules	34 (9.37)	163 (16.84)	0.001	33 (9.88)	30 (8.98)	0.791
Poor Tumor differentiation	281 (77.41)	629 (64.98)	< 0.001	254 (76.05)	253 (75.75)	0.999
Incomplete tumor envelope	30 (8.26)	168(17.36)	< 0.001	29 (8.68)	30 (8.98)	0.999
Tumor diameter > 5 cm	167 (46.01)	427 (44.11)	0.577	153 (45.81)	142 (42.51)	0.436

Values in parentheses are percentages unless stated otherwise; #values are mean(s.d.). AFP, alpha-fetoprotein; ASA, American Society of Anesthesiologists; ALT, alanine aminotransferase; AST, aspartate transaminase; BCLC, Barcelona Clinic Liver Cancer; HBV, hepatitis B virus; HCC, hepatocellular carcinoma; MVI, microvascular invasion

### Comparisons of short- and long-term oncological prognosis

As shown in Table 2, the risk of major morbidity in the elderly group was higher than that in the nonelderly group, both before (14.60% vs. 7.64%, *P* < 0.001) and after (14.07% vs. 6.89%, *P* = 0.004) PSM. The rate of perioperative death in the elderly group was higher than that in the nonelderly group before PSM (4.96% vs. 1.76%, *P* = 0.020). However, this rate was not significantly different between the two groups after PSM (2.40% vs. 1.80%, *P* = 0.787). These results indicated that the elderly group suffered a higher risk of serious postoperative complications, but old age did not increase the probability of perioperative death.

**Table 2:** Comparisons of short- and long-term oncological outcomes between the elderly and the nonelderly groups before and after propensity score matching

Characteristics	Before PSM(n = 1331)		*P*	After PSM(n = 668)		*P*
	Elderly	Nonelderly		Elderly	Nonelderly	
	n = 363	n = 968		n = 334	n = 334	
**Short-term outcome**						
Major morbidity	53 (14.60)	74 (7.64)	< 0.001	47 (14.07)	23 (6.89)	0.004
Perioperative death	18 (4.96)	17 (1.76)	0.002	8 (2.40)	6 (1.80)	0.787
**Long-term outcome**						
Recurrence during follow-up	181 (49.9)	625 (64.6)	< 0.001	172 (51.5)	205 (61.4)	0.010
Median time of recurrence (months)*	27.0 (9.5–50.0)	23.0 (5.9–53.0)	0.784	24.3 (8.0–50.0)	27.0 (7.0-55.3)	0.229
Death during follow-up	246 (67.8)	554 (57.2)	< 0.001	132 (39.5)	152 (45.5)	0.118
Cancer-specific death	140 (38.6)	503 (52.0)	< 0.001	89 (26.6)	131 (39.2)	< 0.001
Noncancer-specific death	106 (29.2)	51 (5.2)	< 0.001	43 (12.9)	21 (6.3)	< 0.001
Median CSS (months) *	39.5 (21.7–60.2)	41.9 (16.2–68.5)	0.073	44.6 (19.3–65.6)	37.5 (18.7–58.7)	0.870
1-year CSS rate, %	91.3	81.5		91.0	86.2	
3-year CSS rate, %	72.1	62.4		71.3	68.8	
5-year CSS rate, %	56.3	51.3		55.9	58.0	

Values in parentheses are percentages unless stated otherwise; * values are median (upper and lower quartile). Continuous variables were compared using the student’s t test and categorical variables were compared using the Fisher’s exact test or the χ2 test, as appropriate. PSM, propensity score matching; CSS, cancer-specific survival

During the follow-up, fewer people experienced recurrence in the elderly group (before PSM:49.9% vs. 64.6%, *P* < 0.010; after PSM: 51.5% vs. 61.4%, *P* = 0.010). However the rates of death during the follow up were similar between the two groups after PSM (39.5% vs. 45.5%, *P* = 0.118). Compared to the nonelderly group, the rate of noncancer-specific death was higher (12.9% vs. 6.3%, *P* < 0.001) in the elderly group, but the rate of cancer-specific death was lower (26.6% vs. 39.2%, *P* < 0.001).

**Fig. 3:**
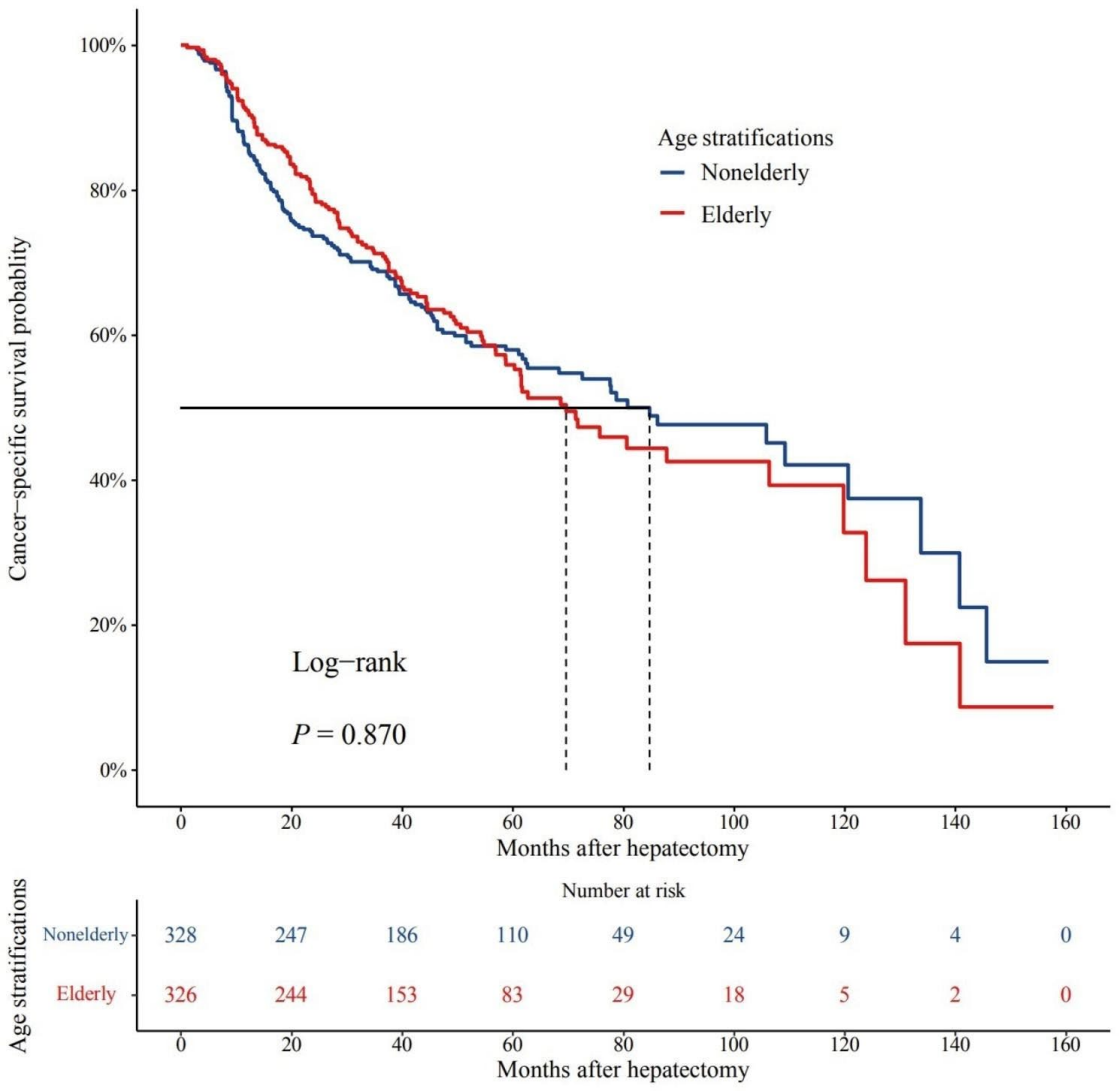
Kaplan–Meier curves comparing the CSS of elderly and nonelderly patients with HCC in the propensity score-matched cohort (P = 0.087)

The CSS between the elderly and non-elderly patients was similiarly before PSM (P = 0.073) (Supplementary Fig. 1). And the 1-, 3- and 5-year CSS rates in the elderly group versus the nonelderly group were 91.0%,71.3%, and 55.9% and 86.2%, 68.8%, and 58.0%, respectively, which were not significantly different between the two groups after PSM (*P* = 0.870) (Fig. 3).

### Univariable and multivariable analyses

Before PSM, the univariate and multivariable analyses both show that the old age itself was independently and significantly associated with major morbidity (Supplementary Table 1) but did not associated with the CSS in multivariable analysis (Supplementary Table 2). After PSM, the univariate analysis showed that among HCC patients undergoing hepatectomy, old age itself was not associated with major morbidity (OR = 0.78, 95% CI: 0.58–1.06, *P* = 0.118) or CSS (HR = 1.02, 95% CI: 0.81–1.29, *P* = 0.873). Then, the multivariate logistic analysis demonstrated that five factors were independent predictors of major morbidity including AFP level (OR = 0.48, 95% CI: 0.23–0.93, *P* = 0.037), Child‒Pugh grade (OR = 2.33, 95% CI: 1.38–5.89, *P* = 0.044), intraoperative blood transfusion (OR = 0.37, 95% CI: 0.16–0.79, P = 0.013), extended hemihepatectomy (OR = 7.46, 95% CI: 4.03–14.07, P = 0.022) and tumour diameter (OR = 1.49, 95% CI: 1.08–2.82, *P* = 0.022) (Table 3). The multivariate Cox-regression analyses showed that the AFP level (OR = 1.92, 95% CI: 1.12–3.29, *P* = 0.018), cirrhosis (HR = 1.34, 95% CI = 1.03–1.74, *P* = 0.030), Child‒Pugh grade (HR = 1.92, 95% CI = 1.12–3.29, *P* = 0.018), macrovascular invasion (HR = 2.00, 95% CI = 1.25–3.21, *P* = 0.004), MVI (HR = 1.20, 95% CI = 1.03–1.58, *P* = 0.034), satellite nodules (HR = 2.48, 95% CI = 1.72–3.57, *P* < 0.001) and tumour diameter (HR = 1.67, 95% CI = 1.28–2.19, *P* < 0.001) were independently and significantly associated with CSS (Table 4).

**Table 3:** Univariate and multivariate logistic regression analyses for predicting postoperative major morbidity after partial hepatectomy for hepatocellular carcinoma after PSM.

Characteristics	comparison	OR (95% CI)	*P*	OR (95% CI)	*P*
Age	Elderly vs. Nonelderly	0.78(0.58–1.06)	0.118		
Sex	male vs. female	1.1(0.74–1.64)	0.642		
ASA score	> 2 vs. ≤ 2	0.56 (0.39–0.8)	0.001	1.01 (0.53–1.87)	0.966
Overweight	Yes vs. No	0.84 (0.6–1.19)	0.331		
Comorbidity	Yes vs. No	0.85 (0.61–1.18)	0.331		
Preoperative AFP level	> 400 ug/L vs.≤ 400 ug/L	1.91 (1.35–2.7)	< 0.001	0.48 (0.23–0.93)	0.037
Preoperative ALT level	> 40 U/L vs. ≤ 40 U/L	0.92 (0.66–1.27)	0.610		
Preoperative AST level	> 40 U/L vs. ≤ 40 U/L	1.45 (1.07–1.98)	0.018	0.96 (0.54–1.68)	0.889
HBV infection	Yes vs. No	1.19 (0.77–1.83)	0.441		
Cirrhosis	Yes vs. No	1.3 (0.95–1.77)	0.096	0.75 (0.4–1.38)	0.358
Portal hypertension	Yes vs. No	1.18 (0.85–1.64)	0.324		
Child‒Pugh grade	B vs. A	3.51 (1.35–9.17)	0.01	2.33 (1.38–5.89)	0.044
BCLC staging	B/C vs. A	2.22 (1.59–3.11)	< 0.001	1.22 (0.78–1.86)	0.472
Open hepatectomy	Yes vs. No	0.39 (0.26–0.6)	< 0.001	1.02 (0.45–2.14)	0.964
Margin width	< 1 cm vs. ≥ 1 cm	1.27 (0.83–1.94)	0.280		
Blood loss	> 600ml vs. ≤ 600ml	2.27 (1.28–4.02)	0.050	1.19 (0.4–3.11)	0.742
Intraoperative blood transfusion	Yes vs. No	1.54 (1.08–2.2)	0.016	0.37 (0.16–0.79)	0.013
Operation duration	> 180 min vs. ≤ 180 min	1.24 (0.89–1.73)	0.200		
Anatomical hepatectomy	Yes vs. No	1.25 (0.92–1.7)	0.152		
Extended hemihepatectomy	Yes vs. No	1.64 (1.09–2.45)	0.017	7.46 (4.03–14.07)	0.022
Occlusion	Yes vs. No	1.28 (0.94–1.75)	0.119		
Macrovascular invasion	Yes vs. No	1.88 (1-3.53)	0.051	1.36 (0.43–3.82)	0.580
MVI	Yes vs. No	1.60 (1.14–2.24)	0.006	1.46 (0.78–2.67)	0.230
Satellite nodules	Yes vs. No	1.92(1.14–3.24)	0.015	0.96 (0.36–2.29)	0.927
Poor Tumor differentiation	Yes vs. No	0.83 (0.58–1.19)	0.310		
Incomplete tumor envelope	Yes vs. No	2.28 (1.32–3.94)	0.003	0.66 (0.22–1.71)	0.421
Tumor diameter	>5cmvs. ≤ 5 cm	1.99 (1.46–2.72)	< 0.001	1.49 (1.08–2.82)	0.022

Values in parentheses are 95% confidence intervals. Those variables found significant at *P* < 0.05 in univariate analyses were entered into multivariate analyses. AFP, alpha-fetoprotein; ASA, American Society of Anesthesiologists; ALT, alanine aminotransferase; AST, aspartate transaminase; BCLC, Barcelona Clinic Liver Cancer; HBV, hepatitis B virus; HCC, hepatocellular carcinoma; MVI, microvascular invasion; CI, confidence interval; OR, odds ratio;

**Table 4:** Univariate and multivariate Cox-regression analyses for predicting cancer-specific survival (CSS) after PSM.

Characteristics	Comparison	HR (95% CI)	*P*	HR (95% CI)	*P*
Age	Elderly vs. Nonelderly	1.02 (0.81–1.29)	0.873		
Male sex	male vs. female	1.09 (0.8–1.48)	0.577		
ASA score	> 2 vs. ≤ 2	0.86 (0.64–1.14)	0.293		
Overweight	Yes vs. No	0.83 (0.63–1.08)	0.168		
Comorbidity	Yes vs. No	0.99 (0.76–1.28)	0.915		
Preoperative AFP level	> 400 ug/L vs. ≤ 400 ug/L	1.68 (1.31–2.16)	< 0.001	1.36 (1.05, 1.77)	0.020
Preoperative ALT level	> 40 U/L vs. ≤ 40 U/L	0.94 (0.74–1.21)	0.380		
Preoperative AST level	> 40 U/L vs. ≤ 40 U/L	1.42 (1.13–1.8)	0.030	1.25 (0.97, 1.61)	0.083
HBV infection	Yes vs. No	1.16 (0.82–1.64)	0.390		
Cirrhosis	Yes vs. No	1.54 (1.22–1.95)	< 0.001	1.34 (1.03, 1.74)	0.030
Portal hypertension	Yes vs. No	1.15 (0.89–1.47)	0.284		
Child‒Pugh grade	B vs. A	1.88 (1.12–3.17)	0.018	1.92 (1.12, 3.29)	0.018
BCLC staging	B/C vs. A	2.33 (1.82–2.97)	< 0.001	1.14 (0.91–1.57)	0.107
Open hepatectomy	Yes vs. No	0.64 (0.45–0.91)	0.014	0.71 (0.49, 1.04)	0.075
Margin width	< 1 cm vs. ≥ 1 cm	1.00 (0.73–1.38)	0.982		
Blood loss	> 600ml vs. ≤ 600ml	1.57 (1.08–2.27)	0.018	1.30 (0.88, 1.92)	0.181
Intraoperative blood transfusion	Yes vs. No	1.7 (1.31–2.21)	< 0.001	1.14 (0.85, 1.52)	0.374
Operation duration	> 180 min vs. ≤ 180 min	1.17 (0.91–1.51)	0.227		
Anatomical hepatectomy	Yes vs. No	1.26 (1-1.6)	0.052	1.32 (0.78, 1.68)	0.067
Extended hemihepatectomy	Yes vs. No	1.43 (1.06–1.93)	0.019	1.00 (0.73, 1.36)	0.978
Occlusion	Yes vs. No	1.22 (0.96–1.55)	0.099	1.05 (0.81, 1.36)	0.721
Macrovascular invasion	Yes vs. No	2.70 (1.76–4.14)	< 0.001	2.00 (1.25, 3.21)	0.004
MVI	Yes vs. No	1.73 (1.34–2.21)	< 0.001	1.20 (1.03, 1.58)	0.034
Satellite nodules	Yes vs. No	2.7 (1.9–3.85)	< 0.001	2.48 (1.72, 3.57)	< 0.001
Poor Tumor differentiation	Yes vs. No	0.92 (0.71–1.21)	0.559		
Incomplete tumor envelope	Yes vs. No	1.53 (1.07–2.19)	0.019	1.42 (0.97, 2.07)	0.071
Tumor diameter	> 5 cm vs. ≤ 5 cm	2.07 (1.63–2.63)	< 0.001	1.67 (1.28, 2.19)	< 0.001

Values in parentheses are 95% confidence intervals. Those variables found significant at *P* < 0.05 in univariate analyses were entered into multivariate analyses. AFP, alpha-fetoprotein; ASA, American Society of Anesthesiologists; ALT, alanine aminotransferase; AST, aspartate transaminase; BCLC, Barcelona Clinic Liver Cancer; HBV, hepatitis B virus; HCC, hepatocellular carcinoma; MVI, microvascular invasion; CI, confidence interval; HR, hazard ratio

## Discussion

The present study utilized a multicentre database to describe and compare the short- and long-term outcomes of elderly and nonelderly HCC patients who underwent R0 resection. The sample size was large with comprehensive data, and the three hospitals selected for the study were large, high-volume hepato-pancreato-biliary surgery centres with accurate and reliable clinical data. PSM was performed before the analysis and comparison, to balance the differences in baseline characteristics between the two datasets and to reduce selection bias [27]. After PSM, the data from the two groups did not differ significantly and were comparable. Multivariate analysis was used to compare the effects of age on the short- and long-term outcomes of HCC patients after surgery. The matched sample size (n = 334) was large, which improved the accuracy and credibility of the results.

The data from both groups showed that the elderly group had a higher probability of serious postoperative complications but the rates of death during the perioperative period and follow-up were similar after PSM. Elderly patients tended to have more comorbid chronic diseases (48.76% vs. 18.29%, *P* < 0.001) and are in worse physical condition. Cirrhosis and its associated portal hypertension (38.64% vs. 33.13%, *P* = 0.014) are more severe in elderly patients than in nonelderly patients, and progression of liver fibrosis results in worse prognosis [28]. In addition, factors associated with cancer in elderly patients such as sarcopenia [29], cachexia [30], and malnutrition [31] also reduce the safety of surgery and survival of elderly patients to some extent. Elderly patients have higher noncancer-specific mortality than nonelderly patients, which adversely affects survival. On the other hand, other studies have shown that younger patients have more malignant and aggressive tumours, a higher probability of recurrence, and a greater tendency towards metastasis [32], consistent with the findings of the present study. Meanwhile, the preoperative evaluation of elderly patients was more rigorous, the surgical treatment of nonelderly patients was more aggressive, and more nonelderly patients with advanced disease (BCLC classification B or C) underwent radical hepatectomy (37.60% vs. 30.03%, *P* = 0.012). This may be related to the llower recurrence and cancer-specific death in the elderly group during follow-up. Meanwhile, we found an interesting result in multivariate analysis that high AFP have a protective role towards morbidity (OR = 0.48), and this may also be related to the above differences. In our study, more nonelderly patients with higher malignancy and later stage underwent hepatectomy and better physical condition meant that this group had a lower probability of major morbidity after hepatectomy than elderly patients. It is well known that AFP is significant risk factor for tumor aggressiveness [33, 34], which may be the reason why high AFP levels seem to have a protective effect on major complications in the present study.

Based on the current patterns of population ageing, the progressive increase in life expectancy has resulted in an increased age of onset of HCC and the number of elderly HCC patients is expected to increase substantially in the future [8]. Thus, prognostic analysis of elderly HCC patients is expected to become a future focus. However, poorer perioperative prognosis and higher noncancer-specific death also indicate that for elderly postoperative HCC patients, not only should the detection and treatment of tumour recurrence be emphasized, but the overall physical condition of the patient warrants attention. Also, the prognosis of the patient can be improved through comprehensive monitoring and treatment. Regarding the age of the patients, especially younger patients (< 35 years old), postoperative monitoring of HCC recurrence must be improved because of the higher probability of recurrence [21].Previous studies have used overall survival (OS) to evaluate long-term oncologic surgical outcomes. However, OS focuses only on overall patient survival and does not consider whether the tumour was the cause of reduced life expectancy, whereas CSS has been used to analyse tumourspecific mortality [35]. In the present study, we considered CSS a more representative and accurate reference indicator for assessing long-term outcomes of surgical treatment with more clinical guiding significance. Elderly patients were found to have similar CSS as nonelderly patients, and radical partial hepatectomy is still considered the preferred and most beneficial treatment option for early-stage elderly HCC patients [36].

The present study has some limitations. First, it was a retrospective clinical study with inherent selection bias, and the use of PSM reduced but did not completely eliminate this bias. Second, the patient population in this study was entirely Chinese with a high proportion of HBV infected patients, which differs from alcoholic liver disease, which is more prevalent in Western countries. These potential confounding factors, which may lead to differences in the study findings, have not yet been verified. Third, the present study confirmed that elderly patients have a higher probability of serious postoperative complications, which affects their overall survival, but the type and severity of the complications were not further investigated [37–39]. Fourth, due to excessive variability, only chronic diseases were included as a separate overall variable in the present study, and no separate analysis of the type and impact of chronic diseases was performed. Although this is a multicentre study, prospective studies are still needed to obtain more reliable conclusions.

## Conclusion

The present study showed that elderly HCC patients undergoing hepatectomy can achieve similar oncological outcomes, to those of nonelderly patients and that partial hepatectomy remains an effective and beneficial treatment option of choice for elderly HCC patients.

### Electronic supplementary material

Below is the link to the electronic supplementary material.

Supplementary Material 1

## Data Availability

The datasets used and analysed during the current study are available from the corresponding author on reasonable request.
