# Supplementary material for: Oncological prognosis and morbidity of hepatectomy in elderly patients with hepatocellular carcinoma: a propensity score matching and multicentre study
Source: BMC Surg. 2023 Oct 24;23:323. doi: 10.1186/s12893-023-02230-0 (PMC10594915; doi:10.1186/s12893-023-02230-0)
Supplement: Supplementary file 1 — Supplementary Material 1 [file 12893_2023_2230_MOESM1_ESM.doc]

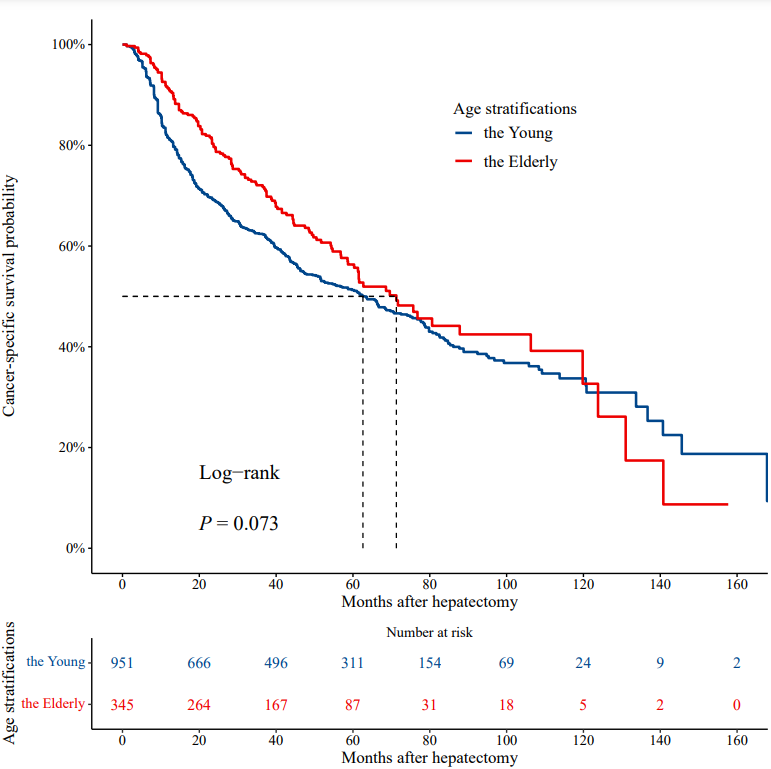


**Supplementary Fig. 1** Kaplan–Meier curves comparing the CSS of elderly and on-elderly patients with HCC in the entire cohort (P = 0.073).

**Supplementary Table 1 Univariate and multivariate logistic regression analyses for predicting postoperative major morbidity after partial hepatectomy for hepatocellular carcinoma beforer PSM.**

| **Characteristics** | **comparison** | **OR (95% CI)** | ***P*** | **OR (95% CI)** | ***P*** |
| --- | --- | --- | --- | --- | --- |
| Age | Elderly vs. Non-elderly | 0.5（0.45-0.73） | <0.001 | 2.68（1.7-4.25） | <0.001 |
| Sex | male *vs.* female | 0.83(0.62-1.12) | 0.223 |  |  |
| ASA scroe | >2 *vs.* ≤2 | 0.57 (0.44-0.74) | <0.001 | 1.18 (0.73-1.88) | 0.487 |
| Overweight | Yes *vs.* No | 0.89 (0.71-1.12) | 0.323 |  |  |
| Comorbidity | Yes *vs.* No | 0.67 (0.52-0.86) | 0.001 | 0.86(0.53-1.37) | 0.527 |
| Preoperative AFP level | >400 ug/L *vs.*≤400 ug/L | 1.81 (1.44-2.28) | <0.001 | 0.49 (0.31-0.78) | 0.003 |
| Preoperative ALT level | >40 U/L*vs.* ≤40 U/L | 1.36 (1.09-1.69) | 0.007 | 0.86 (0.52-1.4) | 0.548 |
| Preoperative AST level | >40 U/L*vs.* ≤40 U/L | 1.54 (1.24-1.91) | <0.001 | 0.95 (0.58-1.55) | 0.843 |
| HBV infection | Yes *vs.* No | 1.1 (0.8-1.51) | 0.569 |  |  |
| Cirrhosis | Yes *vs.*No | 1.12 (0.9-1.39) | 0.308 |  |  |
| Portal hypertension | Yes *vs.*No | 1.03 (0.83-1.29) | 0.764 |  |  |
| Child-Pugh grade | B *vs.*A | 3.85 (2.1-7.05) | <0.001 | 1.76 (0.7-3.97) | 0.196 |
| BCLC staging | B/C*vs.*A | 2.6 (2.06-3.28) | <0.001 | 1.29 (0.8-2.07) | 0.287 |
| Open hepatectomy | Yes *vs.* No | 0.36 (0.27-0.5) | <0.001 | 0.87 (0.47-1.55) | 0.652 |
| Margin width | <1cm*vs.* ≥1cm | 1.51 (1.1-2.09) | 0.012 | 0.48 (0.23-0.89) | 0.029 |
| Blood loss | >600ml*vs.*≤600ml | 2.26 (1.48-3.44) | <0.001 | 1.16 (0.56-2.29) | 0.677 |
| Intraoperative blood transfusion | Yes *vs.*No | 1.43 (1.1-1.85) | 0.007 | 0.4 (0.22-0.69) | 0.001 |
| Operation duration | >180min*vs.*≤180min | 1.42 (1.11-1.82) | 0.006 | 1.45 (0.92-2.35) | 0.122 |
| Anatomical hepatectomy | Yes *vs.*No | 0.94 (0.76-1.16) | 0.566 |  |  |
| Extend hemi hepatectomy | Yes *vs.*No | 2 (1.5-2.67) | <0.001 | 6.7 (4.2-10.8) | <0.001 |
| Occlusion | Yes *vs.*No | 1.4 (1.12-1.74) | 0.003 | 1.25 (0.82-1.92) | 0.302 |
| Macrovascular invasion | Yes *vs.*No | 3.07 (2.06-4.58) | <0.001 | 0.46(0.2-0.98) | 0.055 |
| MVI | Yes*vs.*No | 2 (1.58-2.54) | <0.001 | 1.57 (1.01-2.43) | 0.045 |
| Satellite nodules | Yes*vs.*No | 2.65 (1.92-3.67) | <0.001 | 1.38 (0.79-2.35) | 0.249 |
| Poor Tumor differentiation | Yes*vs.*No | 0.8 (0.64-1.01) | 0.065 | 1.04 (0.67-1.64) | 0.853 |
| Incomplete tumor envelop | Yes*vs.*No | 3.08 (2.21-4.28) | 0.003 | 1.23 (0.69-2.14) | 0.465 |
| Tumor diameter | ＞5cm*vs.* ≤5cm | 2.22 (1.78-2.77) | <0.001 | 0.98 (0.61-1.56) | 0.936 |

Values in parentheses are 95 percent confidence intervals. Those variables found significant at *P*< 0.050 in univariate analyses were entered into multivariate analyses.AFP, alpha-fetoprotein; ASA, American Society of Anesthesiologists; ALT, alanine aminotransferase; AST, aspartate transaminase; BCLC, Barcelona Clinic Liver Cancer; HBV, hepatitis B virus; HCC, hepatocellular carcinoma; MVI, microvascular invasion; CI, confidence interval; OR, odds ratio;

**Supplementary Table 2** Univariate and multivariate Cox-regression analyses for predicting cancer-specific survival (CSS) before PSM.

| **Characteristics** | **Comparison** | **HR (95% CI)** | ***P*** | **HR (95% CI)** | ***P*** |
| --- | --- | --- | --- | --- | --- |
| Age | Elderly vs. Non-elderly | 0.81 (0.67-0.97) | 0.024 | 0.94 (0.76-1.15) | 0.526 |
| Male sex | male vs. female | 0.86 (0.69-1.07) | 0.169 | 0.88 (0.73-1.08) |  |
| ASA score | >2 vs. ≤2 | 0.89 (0.73-1.09) | 0.257 |  |  |
| Overweight | Yes vs. No | 0.87 (0.74-1.03) | 0.098 | 0.99 (0.84-1.18) | 0.932 |
| Comorbidity | Yes vs. No | 0.81 (0.67-0.98) | 0.028 | 0.88 (0.73-1.08) | 0.216 |
| Preoperative AFP level | >400 ug/L vs. ≤400 ug/L | 1.6 (1.36-1.87) | <0.001 | 1.19 (1.01-1.41) | 0.037 |
| Preoperative ALT level | >40 U/L vs. ≤40 U/L | 1.26 (1.07-1.47) | 0.004 | 1.06 (0.87-1.30) | 0.560 |
| Preoperative AST level | >40 U/L vs. ≤40 U/L | 1.52 (1.3-1.78) | <0.001 | 1.12 (0.91-1.37) | 0.279 |
| HBV infection | Yes vs. No | 1.09 (0.86-1.38) | 0.462 |  |  |
| Cirrhosis | Yes vs. No | 1.4 (1.2-1.64) | <0.001 | 1.15 (0.96-1.37) | 0.119 |
| Portal hypertension | Yes vs. No | 0.98 (0.83-1.14) | 0.758 |  |  |
| Child-Pugh grade | B vs. A | 2 (1.49-2.69) | <0.001 | 1.68 (1.24-2.27) | 0.001 |
| BCLC staging | B/C vs. A | 2.58 (2.2-3.02) | <0.001 | 1.38 (1.13-1.68) | 0.001 |
| Open hepatectomy | Yes vs. No | 0.62 (0.48-0.81) | <0.001 | 0.83 (0.63-1.08) | 0.165 |
| Margin width | <1cm vs. ≥1cm | 0.187 (0.93-1.43) | 0.187 |  |  |
| Blood loss | >600ml vs. ≤600ml | 1.46 (1.13-1.88) | 0.004 | 1.07 (0.82-1.40) | 0.629 |
| Intraoperative blood transfusion | Yes vs. No | 1.57 (1.32-1.88) | <0.001 | 1.05 (0.86-1.29) | 0.600 |
| Operation duration | >180min vs. ≤180min | 1.23 (1.02-1.49) | 0.028 | 1.11 (0.91-1.35) | 0.297 |
| Anatomical hepatectomy | Yes vs. No | 0.97 (0.83-1.14) | 0.743 |  |  |
| Extend hemi-hepatectomy | Yes vs. No | 1.62 (1.35-1.96) | <0.001 | 0.90 (0.74-1.11) | 0.337 |
| Occlusion | Yes vs. No | 1.35 (1.15-1.58) | <0.001 | 1.04 (0.88-1.23) | 0.624 |
| Macrovascular invasion | Yes vs. No | 3.15 (2.53-3.94) | <0.001 | 1.57 (1.23-2.02) | <0.001 |
| MVI | Yes vs. No | 2.29 (1.95-2.7) | <0.001 | 1.50 (1.25-1.80) | <0.001 |
| Satellite nodules | Yes vs. No | 2.57 (2.12-3.11) | <0.001 | 1.55 (1.25-1.92) | <0.001 |
| Poor Tumor differentiation | Yes vs. No | 0.91 (0.77-1.07) | 0.237 |  |  |
| Incomplete tumor envelop | Yes vs. No | 1.81 (1.5-2.19) | <0.001 | 1.52 (1.23-1.87) | <0.001 |
| Tumor diameter | >5cm vs. ≤5cm | 2.25 (1.92-2.63) | <0.001 | 1.82 (1.51-2.18) | <0.001 |

Values in parentheses are 95 percent confidence intervals. Those variables found significant at *P*< 0.05 in univariate analyses were entered into multivariate analyses.AFP, alpha-fetoprotein; ASA, American Society of Anesthesiologists; ALT, alanine aminotransferase; AST, aspartate transaminase; BCLC, Barcelona Clinic Liver Cancer; HBV, hepatitis B virus; HCC, hepatocellular carcinoma; MVI, microvascular invasion; CI, confidence interval; HR, hazard ratio.
